# The prevalence of dyslipidemia in patients on hemodialysis: a cross-sectional study from Syria

**DOI:** 10.1097/MS9.0000000000000931

**Published:** 2023-06-05

**Authors:** Yasmeen Kamel Hasan, Mohammad Alsultan, Mohamed Taher Anan, Qussai Hassn, Kassem Basha

**Affiliations:** a Department of Nephrology, Al Assad and Al Mouwasat University Hospitals; b Department of Nephrology, Al Assad University Hospital; c Department of Nephrology, Al Mouwasat University Hospital, Damascus University—Faculty of Medicine, Damascus; d Department of Statics, Aleppo University—Faculty of Sciences, Aleppo, Syria

**Keywords:** atherosclerotic cardiovascular disease score (ASCVD), dyslipidemia, Framingham risk score (FRS), haemodialysis (HD), high-density lipoprotein (HDL), total cholesterol (TC)

## Abstract

Dyslipidemia is an established risk factor for cardiovascular disease (CVD), which is the main cause of mortality among haemodialysis (HD) patients. We investigate the prevalence and characteristics of dyslipidemia in HD patients. Also, we aimed to study the prediction scores; Framingham risk score (FRS), and the atherosclerotic cardiovascular disease risk score; among this population.

**Methods:**

One hundred fifty-three HD patients were enroled in this retrospective cross-sectional study from two HD centres in Syria, from March 2021 to March 2022. Dyslipidemia is considered as follows; hyper-total cholesterol (TC) (≥200 mg/dl), hyper-triglycerides (TG), (≥150 mg/dl), hyper-low-density lipoprotein (LDL) (≥100 mg/dl), hypo-high-density lipoprotein (HDL) (<40 mg/dl), hyper-Non-HDL (≥130 mg/dl).

**Results:**

The most prevalent dyslipidemic parameter was low HDL (72.50%) followed by increased TGs (37.30%). TC, LDL, HDL, and Non-HDL showed differences between males and females (*P*=0.001, 0.015, 0.024, and 0.025; respectively). These parameters were higher in females. History of CVD showed associations with TC, LDL, HDL, and non-HDL (*P*=0.003, 0.007, 0.004, and 0.004; respectively). Additionally, statins showed effects on TC, LDL, and non-HDL (*P*=0.003, 0.0002, and 0.002; respectively); however, no relation with TG and HDL (*P*=0.9 and 0.4). HDL level showed differences in low (7.5%) and intermediate (10%) FRS (*P*=0.01 and 0.028; respectively); however, it did not show a difference in high (20%) FRS (*P*=0.68). The lipids profile did not show differences in different thresholds of atherosclerotic cardiovascular disease scores.

**Conclusion:**

The prevalence of dyslipidemia was high in HD patients in Syria. All lipid parameters except TG showed differences between males and females. Comparisons of lipid parameters with CVD risk stratifications support the need for further studies to prove the benefits of these scores in CVD prediction among the dialysis population.

## Introduction

Highlights

- The most prevalent components of dyslipidemia was low high-density lipoprotein (HDL) followed by increased triglycerides (TGs).
- All lipid parameters except TG showed differences between males and females.
- All lipid parameters except TG showed significant associations with history of cardiovascular disease.
- Lipids profile have no differences in different atherosclerotic cardiovascular disease scores.
- High-density lipoprotein (HDL) showed differences only in low (7.5%) and intermediate (10%) Framingham risk scores (FRS).

Chronic kidney disease (CKD) is a global health burden and constantly increases in prevalence and causes high morbidity and mortality^1,2^. Approximately 2.6 million patients stand on dialysis treatment in the world^3^. Data available on the exact prevalence of CKD in the Arab world is very limited, where most of the data comes from small studies of ~100 patients or less^4^. In all relevant studies of patients with early CKD or end-stage renal disease (ESRD) published to date, cardiovascular disease (CVD) is the predominant cause of this increased mortality, accounting for over 50% of all deaths. Thus, management of risk factors is required in haemodialysis (HD) patients to prevent the acceleration of CVD^5^.

Dyslipidemia is an established risk factor for CVD^6^. Characteristics of HD patients with dyslipidemia are different from the general population. Dyslipidemia in CKD patients is characterized by an inhibition of lipoprotein lipase. Therefore, HD patients are considered to be in a state of impaired catabolism of LDL^7^. Intestinal cholesterol absorption is increased in HD patients^8^. These changes are observed in the clinical setting as an elevated cholesterol level and decreased HDL level^9^.

Several risk stratifications and predictions of CVD events, that are important in CVD prevention, have been published. Such scores use traditional CVD risk factors, such as age, sex, smoking, cholesterol..etc, and nontraditional CVD risk factors, such as the urinary albumin creatinine ratio and the estimated glomerular filtration rate (eGFR). The Framingham risk score (FRS) and the atherosclerotic cardiovascular disease (ASCVD) risk score are among the most prediction scores used worldwide^10,11^.

To the best of our knowledge, several characteristics specific to HD patients have rendered the FRS unsuitable for use in this particular population, meanwhile, there are no studies assessing ASCVD score among dialysis patients^12^. Since there was no previous data from our country in this regard, we proceed with this study to investigate the prevalence and characteristics of dyslipidemia in HD patients. Also, we aimed to study the validity of prediction scores (FRS and ASCVD) among this population.

## Material and methods

A retrospective cross-sectional study was conducted among HD patients from two HD centres in Syria, from March 2021 to March 2022. The study protocol was approved in accordance with the Declaration of Helsinki and aligned with the STROCSS guidelines^13^.

All patients on maintenance HD were enroled. Exclusion criteria were preexisting hepatitis infections and extensive missing data, which meant the loss of relevant medical and drug history. All data were collected by the same nephrology resident and reviewed by other authors. Of a total of 209 patients, 56 patients were excluded, 24 patients had hepatitis B or C infection, and 32 patients due to missing data.

Data from participants included the following: age, sex, time on HD (months), HD sessions per week, residual urinary output (UO), dry weight, systolic blood pressure (SBP), and history of smoke or alcohol consumption. Medical history included a history of diabetes mellitus (DM), hypertension (HTN), CVD, kidney transplantation (KT), and cerebrovascular accident (CVA). Additionally, current drugs were inserted as angiotensin-converting enzyme inhibitors and angiotensin-receptor blockers (ACEIs/ ARBs), diuretics, alpha-blockers, beta-blockers (BB), and statins. Laboratory tests were comprised of urea (Ur), creatinine (Cr), calcium (Ca), albumin (ALB), total protein (TP), sodium (Na), potassium (K), phosphorus (P), uric acid (UA), C-reactive protein (CRP), and parathyroid hormone. Causes of CKD were inserted as follows: Idiopathic, HTN, DM, glomerulonephritis (GN), polycystic kidney disease (PKD), urolithiasis, urinary tract abnormalities, and other causes.

Lipids profile comprised of total cholesterol (TC), triglycerides (TG), low-density lipoprotein (LDL), high-density lipoprotein (HDL), and non-HDL. Blood samples were drawn in the morning between 8 and 10 a.m. after overnight fasting (at least ≥ 12 h). Lipid parameters were measured using an enzymatic colorimetric assay (Hitachi 912 device).

Also, the 10-year risk for CVD, or as known ASCVD and FRS scores were calculated for appropriate participants. Dyslipidemia of each lipids component is considered as follows: hyper-TC (≥200 mg/dl), hyper-TG (≥150 mg/dl), hyper-LDL (≥100 mg/dl), hypo-HDL (<40 mg/dl), hyper-Non-HDL (≥130 mg/dl)^14,15^.

### Statistical analysis

Statistical analysis was performed using the program R 4.02, SPSS version 23.0, and Microsoft Excel 2010. We used nonparametric statistics and parametric statistics such as analysis of variance (ANOVA). The measure of correlation using the Pearson and Spearman correlation coefficient. Descriptive analysis was performed using mean and SD (mean ± SD). Also, we used linear regressions and *t*-tests to study the association between study variables. *P* value<0.05 was considered statistically significant.

## Results

### Demographic characteristics of the study (**Tables 1** – **3**, **4**,**5** and **6**)

**Table 1:** Relationship of lipid profile with sex

	Female *N*=61 (39.8%) Age (44.6 ± 19.48)	Male *N*=92 (60.52%) Age (44.32 ± 16.24)	
Variables	*N* Mean ± SD	*N* Mean ± SD	*P* value
TC (mg/dl)	61 143.91±49.94	88 120.28±35.24	0.001
TG (mg/dl)	60 144.95±130.94	90 136.81±77.70	0.665
LDL (mg/dl)	56 100.58±99.78	81 66.51±28.84	0.015
HDL (mg/dl)	56 36.45±12.36	86 31.62±12.27	0.024
Non-HDL (mg/dl)	56 106.71±49.97	83 89.37±33.46	0.025

HDL, high-density lipoprotein; LDL. low-density lipoprotein; TC, total cholesterol; TG, triglycerides.

**Table 2:** Baseline demographic characteristics of patients according to serum lipid profile

	TC (mg/dl)	TG (mg/dl)	LDL (mg/dl)	HDL (mg/dl)	Non-HDL (mg/dl)	
	Mean ± SD	Mean ± SD	Mean ± SD	Mean ± SD	Mean ± SD	
	129.95 ± 43.32	140.06 ± 102.03	80.43 ± 69.27	33.52 ± 12.49	96.36 ± 41.63	
	*P*					Mean ± SD or *N* (%)
Age (years)	0.94	0.29	0.93	0.29	0.86	44.5±17.53
Time on HD (months)	0.41	0.08	0.53	0.68	0.17	41.29±48.29
UO (ml/d)	0.24	0.13	0.59	0.14	0.58	559.71±816.07
Dry weight (kg)	0.50	0.19	0.72	0.14	0.16	66.36±25.84
Transplant	**0.011**	0.324	0.140	0.565	**0.032**	19 (12.4)
Trans drug	**0.001**	0.558	**0.001**	0.709	**0.006**	9 (5.8)
HTN	0.052	0.740	0.083	0.642	0.058	111 (73)
DM	0.533	0.587	0.669	0.842	0.643	24 (15.6)
CVD	**0.003**	0.081	**0.007**	**0.004**	**0.004**	24 (15.6)
CVA + CVD	**0.004**	0.064	**0.010**	**0.029**	**0.003**	27 (17.6)
Statins	**0.003**	0.907	**0.0002**	0.471	**0.002**	21 (13.7)
Diuretics	0.420	0.430	0.544	0.508	0.557	35 (22.8)
BB	0.134	0.142	0.071	0.910	0.141	41 (26.7)
ACEi/ARBs	0.218	**0.012**	0.364	0.211	0.062	21 (13.7)
a Blockers	0.055	0.515	**0.021**	0.494	**0.029**	10 (6.5)
Smoking	0.767	0.613	0.547	0.466	0.796	35 (22.8)
Alcohol	0.954	0.583	0.539	0.051	0.421	5 (3.2)
SBP (mmHg)	0.93	0.16	0.50	0.79	0.83	138.84 ± 26.10

The significant P.values in Bold

ACEi/ARBs, angiotensin-converting enzyme inhibitors and angiotensin-receptor blockers; BB, beta-blockers; CVA, cerebrovascular accident; CVD, cardiovascular Disease; DM, diabetes mellitus; HD, haemodialysis; HDL, high-density lipoprotein; HTN, hypertension; LDL. low-density lipoprotein; SBP, systolic blood pressure; TC, total cholesterol; TG, triglycerides; UO, urinary output.

**Table 3:** Comparison of lipid parameter between number of HD sessions per week

	TC	TG	LDL	HDL	Non-HDL
HD sessions (number per week)	*P* value	*P* value	*P* value	*P* value	*P* value
2/ w *N*=104 (68.4%)					
1/ w *N*=5	0.88	**0.02**	0.84	0.61	0.71
3/ w *N*=11	0.051	**0.01**	0.62	0.87	**0.01**

The significant P.values in Bold.

HDL, high-density lipoprotein; LDL. low-density lipoprotein; TC, total cholesterol; TG, triglycerides.

**Table 4:** The relationship of laboratory tests with lipid parameters

	Chol	TG	LDL	HDL	Non-HDL	
Variable	*P*					Mean ± SD
Cr (mg/dl)	0.37	0.08	0.91	0.70	0.13	10.17±3.74
Ur (mg/dl)	0.63	0.09	0.84	0.11	0.44	156.69±70.34
TP (mg/dl)	**0.02**	0.14	0.14	0.49	**0.02**	6.28±1.01
ALB (mg/dl)	0.29	0.72	0.97	0.13	0.70	3.77±1.43
UA (mg/dl)	0.27	**0.01**	0.08	**0.04**	**0.05**	6.88±2.59
CRP (mg/dl)	0.09	0.30	0.13	0.08	0.56	7.58±13.78
Na (mEq/l)	0.74	0.94	0.62	0.70	0.66	136.7±5.64
K (mEq/l)	0.85	0.91	0.41	0.61	0.82	5.04±1.09
Ca (mg/dl)	0.09	0.85	**0.01**	**0.006**	0.35	8.12±1.18
P (mg/dl)	0.99	0.40	0.58	0.16	0.69	6.69±11.13
PTH (pg/ml)	0.99	0.06	0.58	0.19	0.77	705.5±816.5

The significant P.values in Bold.

ALB, albumin; Ca, calcium; Chol, cholesterol; Cr, creatinine; CRP, C-reactive protein; HDL, high-density lipoprotein; K, potassium; LDL. low-density lipoprotein; NA, sodium; P, phosphorus; PTH, parathyroid hormone; TG, triglycerides; TP, total protein; UA, uric acid; Ur, urea.

**Table 5:** Correlations for laboratories of significant relations with lipid parameters

	Chol	TG	LDL	HDL	Non-HDL
TP	0.23	—	—	—	0.24
UA	—	0.25	—	0.22	0.21
Ca	—	—	0.23	0.23	—

Ca, calcium; Chol, cholesterol; HDL, high-density lipoprotein; LDL. low-density lipoprotein; TG, triglycerides; TP, total protein; UA, uric acid.

**Table 6:** Percentage of dyslipidemia and the hazard ratio of dyslipidemia with time on HD

	Definition (mg/dL)	Number	Percentage (%)	Total number
Hyper-TC	≥200	10	6.70	149
Hyper-TG	≥150	56	37.30	150
Hyper-LDL	≥100	28	20.40	137
Hypo-HDL	<40	103	72.50	142
Hyper-Non-HDL	≥130	23	16.50	139

HD, haemodialysis; HDL, high-density lipoprotein; LDL. low-density lipoprotein; TC, total cholesterol; TG, triglycerides.

**Figure 1:**
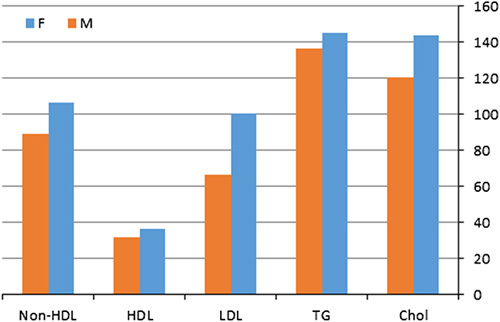
Lipid distribution between M (males) and F (females). HDL, high-density lipoprotein.

Males comprised 92 (60.52%) of patients with mean age (44.6 ± 19.48 years). 61 (39.8%) of patients were females with mean age (44.32 ± 16.24 years) (Table 1). Of lipid parameters; TC, LDL, HDL, and non-HDL showed statistically significant differences between males and females (0.001, 0.015, 0.024, and 0.025; respectively). TG did not show a significant difference between genders (*P*=0.665) (Table 1) (Fig. 1). Also, higher levels of lipids tend to be in females than males.

Causes of CKD are as follows; idiopathic (*n*=51; 34%), HTN (*n*=34; 22%), DM (*n*=13; 8%), GN and urolithiasis (*n*=8; 5% for each), PKD and urinary tract abnormalities (*n*=10; 7% for each), and other conditions (*n*=18; 12%). The most common comorbidity is HTN 111 (73%), and only 21 patients (13.7%) used statins.

**Figure 2:**
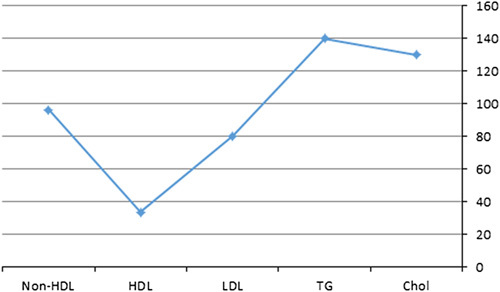
Mean serum levels of lipid profile. Chol, cholesterol; HDL, high-density lipoprotein; LDL. low-density lipoprotein; TG, triglycerides.

Mean serum levels of the lipid profile were shown in Table 2 and Figure 2. TC had statistically significant relations with the history of KT, use of transplant drugs, CVD, CVD plus CVA, and use of statins. TG had only a statically significant relation with the use of ACEi/ARBs. LDL had statistically significant relations with the use of transplant drugs, CVD, CVD plus CVA, statins, and α blockers. HDL had statistically significant relations with CVD, and CVD plus CVA. Non- HDL had statically significant relations with a history of KT, use of transplant drugs, CVD, CVD plus CVA, statins, and α blockers.

A comparison of lipid parameters with HD sessions per week showed in Table 3. Most patients receive twice HD sessions per week (*n*=104, 68.4%). TG showed significant differences between twice HD sessions per week compared with once and thrice HD sessions per week. Non-HDL had a significant difference only between twice HD sessions per week and thrice HD sessions per week.

Studying the relations of laboratory tests with lipid parameters showed in Tables 4 and 5. TP showed a statistically significant relation with TC and non-HDL (*P*=0.02 for each) (Table 4). UA level showed statically significant effects on TG, HDL, and non-HDL (*P*=0.01, 0.04, and 0.05; respectively) (Table 4). Ca level showed statistically significant effects on LDL and HDL (*P*=0.01 and 0.006; respectively). These laboratory tests showed weak correlations with lipid profiles (Table 5).

**Figure 3:**
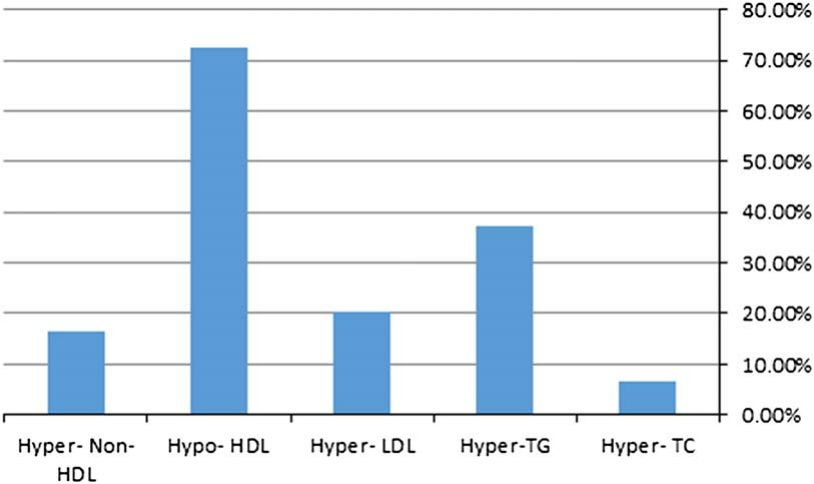
The prevalence of dyslipidemia. HDL, high-density lipoprotein; LDL. low-density lipoprotein; TC, total cholesterol; TG, triglycerides.

The most prevalent component of dyslipidemia was low HDL (72.50%) followed by increased TGs (37.30%) (Table 6) (Fig. 3).

### Lipid differences in ASCVD and FRS scores (**Tables 7** and **8**)

**Table 7:** Regression analysis of lipids with ASCVD score; *N*=59

	Low threshold		
Lipids	<7.5%	≥7.5%	*P* value
TC (mean ± SD)	134.01±43.14	128.18±38.21	0.58
TG (mean ± SD)	126.24±69.25	171.30±165.74	0.18
LDL(mean ± SD)	106.81±136.43	70.27±32.49	0.18
HDL (mean ± SD)	32.44±11.08	31.49±14.87	0.78
Non-HDL (mean ± SD)	101.65±38.97	96.53±37.69	0.61
	**Intermediate threshold**		
	**<10%**	**≥10%**	***P* value**
TC (mean ± SD)	130.5±43.1	131.4±37.6	0.94
TG (mean ± SD)	153.7±160.5	143.7±59.1	0.78
LDL(mean ± SD)	95.8±121.6	74.6±35.02	0.42
HDL (mean ± SD)	33.04±12.8	30.7±13.9	0.53
Non-HDL (mean ± SD)	97.5±40.8	100.3±35.1	0.78
	**High threshold**		
	**<20%**	**≥20%**	***P* value**
TC (mean ± SD)	132.77±41.84	124.05±34.87	0.45
TG (mean ± SD)	156.91±143.87	123.89±51.46	0.21
LDL(mean ± SD)	92.82±107.44	60.82727±22.49	0.06
HDL (mean ± SD)	31.03±12.14	35.1±16.48	0.41
Non-HDL (mean ± SD)	101.79±39.46	88.56±31.73	0.22

ASCVD, atherosclerotic cardiovascular disease; HDL, high-density lipoprotein; LDL. low-density lipoprotein; TC, total cholesterol; TG, triglycerides.

**Table 8:** Comparison of lipids with Framingham Risk Score; *N*=76

	Low threshold		
Lipids	<7.5%	≥7.5%	*P* value
TC (mean ± SD)	149.78±64.25	129.98±40.61	0.24
TG (mean ± SD)	141.61±117.31	147.89±130.39	0.84
LDL(mean ± SD)	87.33±45.11	76.66±37.88	0.37
HDL (mean ± SD)	39.59±12.01	31.12±12.24	*0.01*
Non-HDL (mean ± SD)	111.86±63.13	98.4±38.52	0.40
	**Intermediate threshold**		
	**<10%**	**≥10%**	***P* value**
TC (mean ± SD)	144.37±55.16	130.12±42.75	0.25
TG (mean ± SD)	136.12±101.99	153.61±140.69	0.542
LDL(mean ± SD)	85.68±44.41	75.80±36.95	0.337
HDL (mean ± SD)	37.43±11.49	30.89±12.75	*0.028*
Non-HDL (mean ± SD)	106.26±54.70	99.17±40.22	0.563
	**High threshold**		
	**<20%**	**≥20%**	***P* value**
TC (mean ± SD)	138.63±51.01	131.65±38.15	0.51
TG (mean ± SD)	156.17±150.42	125.66±54.11	0.21
LDL(mean ± SD)	82.47±42.91	73.11±32.66	0.29
HDL (mean ± SD)	33.63±11.64	32.27±14.55	0.68
Non-HDL (mean ± SD)	104.75±49.50	95.75±37.05	0.37

HDL, high-density lipoprotein; LDL. low-density lipoprotein; TC, total cholesterol; TG, triglycerides.

ASCVD (Table 7) score was calculated for appropriate participants (*n*=59; 40.67% of the total sample). Studying the differences between lipids profiles depending on ASCVD thresholds were obtained. The lipids profiles did not show statistically significant differences in low (7.5%), intermediate (10%), and high (20%) risk thresholds of ASCVD.

FRS score (Table 8) was calculated for appropriate participants (*n*=76; 49.67% of the total sample). Studying differences between lipids profiles depending on FRS thresholds was obtained. There were no statistically significant differences in TC, TG, LDL, and non-HDL in FRS scores (7.5%, 10%, 20%). On the other hand, HDL showed significant differences in low (7.5%) and intermediate (10%) risk thresholds of FRS (*P*=0.01 and 0.028; respectively); however, HDL did not show a statistically significant difference (*P*=0.68) in high-risk threshold (20%) of FRS.

## Discussion

Lipid metabolism alterations are frequent in dialysis patients. The classic observation of dyslipidemia in HD patients is characterized by increased TGs, decreased HDL, and usually normal or only slightly elevated total TC and LDL. Approximately 20–40% of HD patients have been estimated to have elevated TGs and reduced HDL^9,14^. Also, in a multicenter study including 25 HD centres in Catalonia, the most frequent lipid alterations recorded were decreased HDL, followed by increased TGs^13^. This study observed a significant elevation of LDL, TC, and HDL in females compared to males; however, TG did not differ between gender^14^. On the other hand, a large observational study from 2002 to 2017 in Korea reported an increased prevalence of dyslipidemia from 18.9 to 86.7%. Explaining this sharp increase by the new KDIGO guideline for dyslipidemia that was released in 2013^16,17^.

Our study corresponds with previous studies on the high prevalence of dyslipidemia among the HD population. Low HDL and elevated TGs, estimating 72.50% and 37.30%; respectively, account for the most common dyslipidemic parameters. A sex variation in lipid parameters was observed in this sample. Dyslipidemic components, including increased levels of TC, TG, LDL, and non-HDL, were prominent in females compared with males. Although HDL levels were reduced in both sex, mean levels of HDL were higher among females (36.45 vs. 31.62). All lipid parameters except TG showed statistically significant differences between gender (Tables 1 – 3).

It was estimated that more than 80% of the global burden of CVD occurred in low-income and middle-income countries and has been continuously rising^18,19^. Chronic HD patients showed a high incidence and prevalence of CVD with a strong association between dyslipidemia and accelerated atherosclerosis^14,20^. HD- patients with a history of ischaemic heart disease had higher TGs and lower HDL concentrations^14,20^. TC level was significantly positively associated with CVD^14,20,21^. Also, higher LDL and non-HDL levels were associated with a higher risk of CVD events and mortality among the HD population^14,20,22^.

In the current study, the history of CVD was observed in 24 patients (15.6%), and CVD including CVA in 27 patients (17.6%). History of CVD showed statistically significant associations with lipid parameters (TC, LDL, HDL, and non-HDL; *P*< 0.05); however, TG did not show associations with a history of CVD or CVA.

Risk stratifications and predictions of CVD events are important in CVD prevention and have been widely used in the general population, such as the FRS and the more recent ASCVD risk score^10,11^. The FRS, which includes TC or LDL, and HDL^23,24^, is the most commonly used for predicting 10-year incidence of CV events in the general population^25^. Previous studies have noted that the FRS cannot adequately predict the risk of CV events among HD patients^26^, even after incorporating HD-specific risk factors such as metabolic syndrome status, and albuminuria^27^. Further, several risk factors included in the FRS are not necessarily risk factors for HD patients. For example, while high TC and LDL levels are reported to be risk factors for CV events in the general population, low TC and LDL levels have been implicated in the risk of death due to CV events in HD patients paradoxically^21,28^. Furthermore, the FRS doesn’t include HD-specific risk factors, such as mineral metabolism^29^, anaemia^30^, and malnutrition^31^, which all have been known as risk factors for CV events. These discrepancies between the general population and HD patients render the FRS inappropriate for use in HD patients^12^.

In thresholds of FRS score, we found differences in HDL levels in low (7.5%) and intermediate (10%) risk scores; however, HDL did not show a statistically significant difference in high FRS score (20%). Also, TC did not show a difference in all FRS scores. Meanwhile, a meta-analysis indicated that higher levels of HDL showed a harmful effect on cardiac death in the pooled analysis results from patients undergoing HD^32^. Further studies should be applied to specify the level of HDL in the prevention of CVD or mortality among the dialysis population.

To the best of our knowledge, there were no studies assessing the validity of ASCVD score among dialysis patients; however, previous reports assessed and improved the prediction of this score among CKD patients who did not require dialysis^19,33^. Since we did not find differences in lipid levels (TC, TG, LDL, HDL, and non-HDL) in our HD participants based on different ASCVD score cut-offs. Moreover, both scores; ASCVD and FRS, share the same lipid parameters. So, the same weakness domains of FRS in dialysis patients could be applied to ASCVD. These results mandate further studies to evaluate the benefits of FRS and ASCVD in the prediction of CV events among the dialysis population. Also, these scores could be improved the prediction by substitution of lipid parameters or incorporating HD-specific risk factors.

Statins showed significant effects on TC, LDL, and non-HDL; however, no relation with TG and HDL in our study. Resembling previous studies, statins affected the lipid profile in patients undergoing dialysis despite a lack of benefits in reducing CVD events^14,34,35^. Statins had significant reduction effects on TC, LDL, and TG but not HDL^14,35^. Another study reported a significant reduction in LDL, TC, and TG levels with a modest increase in HDL by rosuvastatin^34^. Also, data showed that both simvastatin and atorvastatin effectively reduced non-HDL levels^36^.

Numerous studies across a spectrum of ASCVD risk showed that a reduction in LDL with statin therapy is associated with an overall reduction in major vascular and coronary events in the general population^37^. Although these results were uncertain in HD patients based on data from three large-scale randomized controlled trials^34,35,38^. Also, assessing outcomes 7.4 years beyond the randomization period of the 4D study (atorvastatin vs. placebo), were showed no differences in a composite CV outcome^39^.

Currently under the new cholesterol guidelines for lipid management for adults with advanced CKD that requires dialysis treatment published in 2018 by the American College of Cardiology (ACC)/ the American Heart Association (AHA), 2019 of European Society of Cardiology (ESC)/ European Atherosclerosis Society (EAS), and the last KDIGO guidelines (2013), did not recommend the initiation of a statin; however, it is reasonable to continue statins if the patient is currently on LDL-lowering therapy^17,39–41^.

Limitations of the presented study were the relatively small sample size restricted retrospectively to two dialysis centres, and the cross-sectional design that did not determine causal relationships. Also, other risk factors of CVD could not be included such as malnutrition which is represented with low TC levels. Nevertheless, all lipid parameters were collected and studied with extensive demographic and laboratory data. Also, the comparison of lipid profiles between several thresholds of CVD risk scores (ASCVD and FRS) provides some novel insights for future studies to consider risk factors other than lipids when these scores could apply in dialysis patients.

## Conclusion

The prevalence of dyslipidemia was high in HD patients in Syria. All lipid parameters except TG showed differences between males and females. The lipids profile did not show significant differences in all risk scores of ASCVD. Only HDL showed differences in low (7.5%) and intermediate (10%) FRS scores. These comparisons of lipid parameters with CVD risk stratifications support the need for further studies to prove the benefits of these scores in CVD prediction among the dialysis population.

## Ethical approval

The study protocol was approved by the Research Ethics Committee of Damascus University in the number (3735), in accordance with the Declaration of Helsinki, and in line with the STROCSS criteria.

## Consent

Written informed consent was obtained from patients for publication of this article and any accompanying images.

## Source of funding

None declared.

## Author contributions

Y.K.H. wrote the manuscript, literature search, and submitted the article. M.A. wrote and correct the manuscript and literature search. M.T.A. did data analysis, wrote and explain the study results. K.B. and .Q.H. made study corrections and supervisor of the research.

## Conflicts of interest disclosure

None declared.

## Research registration unique identifying number (UIN)

1. Name of the registry: OSF Preregistration.
2. Unique Identifying number or registration ID: https://osf.io/xutc8
3. Hyperlink to your specific registration (must be publicly accessible and will be checked): https://archive.org/details/osf-registrations-xutc8-v1.

## Guarantor

Dr. Yasmeen Kamel Hasan.

## Data availability

The data are available from the corresponding author upon reasonable request.

## Provenance and peer review

Not commissioned, externally peer-reviewed
